# Morphological and Phylogenetic Characterization of Three Novel *Thaxterogaster* (*Cortinariaceae*) Species from China with an Emphasis on Their Subtropical Distribution

**DOI:** 10.3390/jof9111058

**Published:** 2023-10-28

**Authors:** Meng-Le Xie, Na Feng, Wen-Fei Lin, Wen-Ying Su, Yi Li, Zhen-Quan Yang

**Affiliations:** 1 School of Food Science and Engineering, Yangzhou University, Yangzhou 225127, China; xiemengle@yzu.edu.cn (M.-L.X.); mz120222073@stu.yzu.edu.cn (N.F.); 2 Institute of Edible and Medicinal Fungi, College of Life Science, Zhejiang University, Hangzhou 310058, China; wenhui33@126.com; 3 Lianyungang Academy of Agricultural Sciences, Lianyungang 222006, China; suwenying1026@126.com

**Keywords:** *Basidiomycota*, edible fungi, ITS, new species, taxonomy

## Abstract

Three new phlegmaciod species of *Thaxterogaster*, *T. borealicremeolinus*, *T. rufopurpureus*, and *T. sinopurpurascens* spp. nov., from subtropical China were described based on their morphological characteristics and molecular data. *Thaxterogaster borealicremeolinus* belongs to the sect. *Cremeolinae* and differs from the other species in this section in its larger basidiospores and its habitat in the Northern Hemisphere associated with *Quercus* sp. trees. *Thaxterogaster rufopurpureus* and *T. sinopurpurascens* belong to sect. *Purpurascentes*, in which *T. rufopurpureus* is characterized by a pileus with a reddish-brown coloration when mature and a clavate stipe, while *T. sinopurpurascens* is characterized by a violet basidiomata, except for a greyish orange to brown pileus, the distinctly marginate bulb of its stipe, and its distribution in subtropical China. The phylogenetic analyses were performed based on nrITS, and detailed descriptions of the new species are provided herein.

## 1. Introduction

*Thaxterogaster* Singer was proposed as a sequestrate genus in *Gasteromycetes* Fr., *Secotiaceae* Tul. & C. Tul. [1]. Later, this genus was changed to *Cortinariaceae* R. Heim [2]. *Thaxterogaster* was used by taxonomists as an independent genus until the early part of this century when molecular phylogenetic studies showed that this genus was nested within *Cortinarius* (Pers.) Gray and was thus proposed as a synonym of *Cortinarius* [3]. Recently, the genus *Cortinarius* was split into ten genera based on multi-gene sequence and genomic data; *Thaxterogaster* was emendated and, as a genus, separated from other genera of *Cortinariaceae*, which followed the Shenzhen Code (Art. 11.4 of the Code) [4].

The currently accepted concept of the genus *Thaxterogaster* is a morphologically diverged group of fungi comprising the phlegmacioid and myxacioid agaric species that were previously classified within *Cortinarius* and some sequestrate species in early genera like *Thaxterogaster* and *Gigasperma* E. Horak [4]. Currently, the genus *Thaxterogaster* is recognized as bihemispherical, with more than 170 species described all over the world [5], and it includes six subgenera and 23 sections [4].

*Thaxterogaster* species usually form obligate ectomycorrhizal associations with *Fagaceae*, *Betulaceae*, *Tilia*, and *Pinaceae* trees in the Northern Hemisphere and *Nothofagaceae* in the Southern Hemisphere [4]. Species in *Thaxterogaster* may have important economic value. For example, some species like *T. multiformis* (Fr.) Niskanen & Liimat. and *T. purpurascens* (Fr.) Niskanen & Liimat. are delicious edible fungi and widely consumed in China [6], and several species, such as *T. turmalis* (Fr.) Niskanen & Liimat., contain certain chemical components with antitumor activity [7].

Currently, there are only 22 known *Thaxterogaster* species in China, but before Liimatainen et al. emendated this genus, these species were recorded in the genus *Cortinarius* s.l. [8,9,10,11]. In the past, the research on *Cortinariaceae* species focused on the temperate zones of China and its Tibetan Plateau, publishing a series of new species [12,13,14,15] but paying less attention to *Cortinariaceae* species in the subtropical and tropical regions of China. Recently, we collected some specimens of *Cortinariaceae* from subtropical China, some of which are new and rare species. Here, we describe three *Thaxterogaster* species collected from subtropical China and establish them as new to science based on morphological and ecological evidence and phylogenetic results.

## 2. Materials and Methods

### 2.1. Specimens and Morphological Description

Specimens included in this study were collected in subtropical China in spring and early summer. Basidiomata were photographed in the field and dried in an oven at about 50 °C. All specimens were deposited in the Herbarium Mycologicum, Academiae Sinicae, Institute of Microbiology, Beijing, China (HMAS).

The macroscopic characteristics were described based on fresh basidiomata. The basidiospores, basidia, sterile cells, and pileipellis were observed in a 5% potassium hydroxide water solution, Congo Red, and Melzer’s reagent under a light microscope. Thirty mature basidiospores were measured per specimen, and the Q (L/W ratio) values were calculated for all basidiospores. X_av._ and Q_av._ indicate the average values of basidiospores in every specimen. Twenty basidia and sterile cells per specimen were measured from the pieces of lamellae. The pileipellis structures were studied from radial sections situated halfway from the pileus center. The photos of basidiospores and pileipellis were taken with an EMCOMOS Camera HY-2000W.

### 2.2. Molecular Phylogeny

The genomic DNA was extracted from the dried specimens using the standard cetyltrimethylammonium bromide (CTAB)-chloroform method with a few modifications [16,17]. The primers ITS1F and ITS4 were used to amplify the ITS region [18,19]. The PCR procedures are as follows: initial denaturation at 95 °C for 5 min, followed by 35 cycles at 95 °C for 30 s, 48 °C for 30 s, and 72 °C for 1 min and a final extension of 72 °C for 10 min. Sequencing was performed by Beijing Tsingke Biotech (Beijing, China) Co., Ltd. All the newly generated sequences were submitted to GenBank, and BLASTn was run in NCBI to select the closely related species for the phylogenetic analyses. We also selected sequences from the other two sections, i.e., sect. *Multiformes* Niskanen & Liimat. and sect. *Riederorum* Niskanen & Liimat., for phylogeny reconstruction because they are also members of *Thaxterogaster* [4]. Two *Phlegmacium* (Fr.) Wünsche species, *P. cyanites* (Fr.) M.M. Moser and *P. boreicyanites* (Kytöv., Liimat., Niskanen & A.F.S. Taylor) Niskanen & Liimat., were chosen as an out-group. All sequences used in the phylogenetic analyses are shown in Table 1.

ITS dataset used for phylogenetic analyses was aligned and manually adjusted using BioEdit 7.1.3.0 [20]. Phylogenetic analyses were performed using Bayesian Inference (BI) and Maximum Likelihood (ML) methods. The best model (GTR + I + G) for BI analysis was chosen according to the Akaike information criterion (AIC) using the software MrModeltest 2.3 [21]. Bayesian analysis was performed with MrBayes 3.2.6 [22]. Two independent analyses of two parallel runs and four chains were carried out for 10,000,000 generations, sampling every 1000 generations. The first 25% of the trees were discarded as burn-in. The ML analysis was implemented using RAxML 8.2.12 in raxmlGUI with a rapid bootstrapping algorithm of 1000 replicates [23,24]. Default parameters of the GTRGAMMA model were used in the ML analysis.

## 3. Results

### 3.1. Molecular Phylogeny

**Figure 1:**
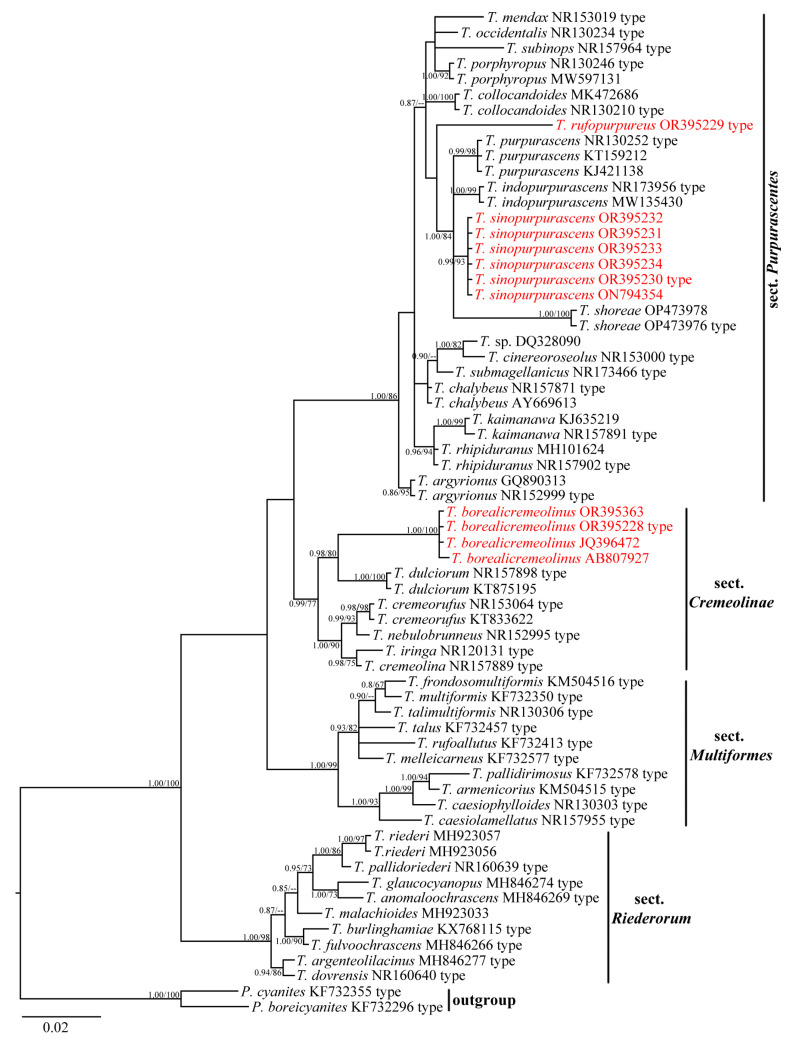
BI tree inferred from ITS sequences. The tree is rooted in *Phlegmacium* species. Bayesian posterior probabilities (≥0.80) and ML bootstrap values (≥60%) are shown on each branch (BPP/ML). New species are indicated by red font.

A total of 65 sequences, including 41 from type materials, were used in the phylogenetic analyses, representing 44 species and one undescribed species. The BI and ML trees showed similar topologies, and the BI tree was selected as a representative example (Figure 1). The Bayesian posterior probabilities (BPP) ≥ 0.80 and ML bootstrap values (ML) ≥ 60% are shown on the branches (BPP/ML).

*Thaxterogaster* species were clustered into four clades in the phylogenetic tree, representing four instinct sections, i.e., sect. *Cremeolinae* (Soop) Niskanen & Liimat., sect. *Multiformes*, sect. *Purpurascentes* (Kühner & Romagn. ex Brandrud & Melot) Niskanen & Liimat., and sect. *Riederorum*, respectively. The sect. *Purpurascentes* clade (BPP/ML = 1.00/86%) included fourteen known species, two novel species, and one undescribed species. *Thaxterogaster rufopurpureus* nom. prov. could be separated from all the other species in this section. For another new species, *T. sinopurpurascens* nom. prov., our five collections together with HKAS 12252 (recorded as *T. purpurascens*) were clustered into a distinct lineage with strong statistical support (BPP/ML = 0.99/93%), which was found to be closely related to *T. purpurascens* and *T. indopurpurascens* (Dima, Semwal, Brandrud, V. Papp & V.K. Bhatt) A. Ghosh, D. Chakr., K. Das & Vizzini and *T. shoreae* A. Ghosh, D. Chakr., K. Das & Vizzini. In the sect. *Cremeolinae* clade (BPP/ML = 0.99/77%), six species clustered, in which *T. borealicremeolinus* nom. prov. (BPP/ML = 1.00/100%) formed a sister relationship (BPP/ML = 0.98/80%) with *T. dulciorum* (Soop) Niskanen & Liimat. In addition, the species of sect. *Multiformes* clade (BPP/ML = 1.00/99%) clustered into two subclades, and eight species of sect. *Riederorum* formed a separated clade with high statistical support (BPP/ML = 1.00/98%).

### 3.2. Taxonomy

**Figure 2:**
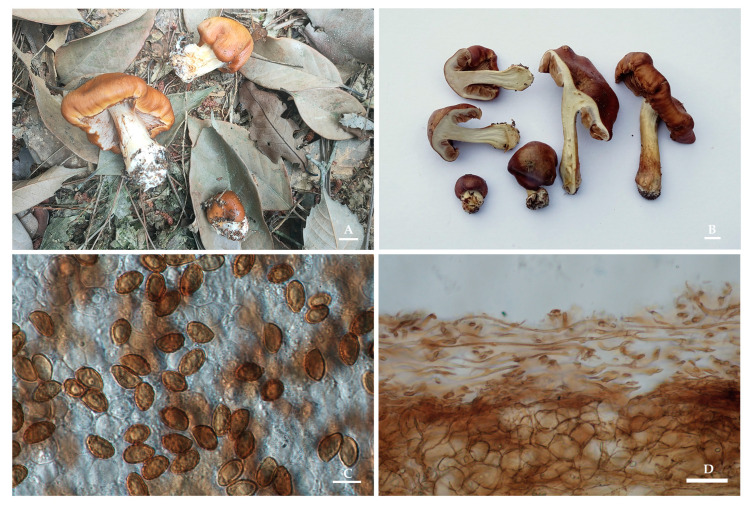
*Thaxterogaster borealicremeolinus* (HMAS 287398). (**A**,**B**) Basidiomata; (**C**) Basidiospores; (**D**) Pileipellis in 5% KOH. Bars: (**A**,**B**) = 1 cm; (**C**) = 10 μm; (**D**) = 40 μm.

*Thaxterogaster borealicremeolinus* M.L. Xie & Yi Li, sp. nov. Figure 2.

Fungal Names: FN 571635.

Etymology: The epithet ‘*borealicremeolinus*’ refers to the affinity of other species of the sect. *Cremeolinae* but with a Northern Hemisphere distribution.

Holotype: CHINA. Zhejiang Province, Xinchang County, in mixed forests, 13 May 2023, *Chao-Yong Liang*, HMAS 287398.

Description: Pileus 3–6.7 cm in diam., hemispherical at first, later plano-convex, viscid, yellowish-brown to reddish-brown, the margin persistently wavy. Lamellae emarginated, moderately crowded, whitish at first, greyish-brown when mature, edges even. Stipe 2.5–6.5 cm long, 0.7–1.1 cm thick at the apex, cylindrical with an expanded base (somewhat marginate bulb), up to 1.6 cm, whitish, surface with yellowish fibrils, basal mycelium white. Universal veil yellowish. Context thick, white.

Basidiospores (10)11–12.5(13) × 6–7.5(8) μm, X_av._ = 11.5 × 7.1 μm, Q = 1.43–1.85, Q_av._ = 1.64, ellipsoid to subamygdaloid, moderately to somewhat strongly verrucose, indextrinoid. Basidia (18)25–38.5 × (7)10–14 μm, clavate, two- or four-spored, thin-walled, hyaline in 5% KOH. Lamellar edges fertile, with clavate sterile cells, 15–28.5 × 5–10.5 μm, thin-walled, hyaline in 5% KOH. Pileipellis duplex: epicutis of gelatinous, 80–150 µm thick, hyphae 2.5–10 μm wide, yellowish to lightly brown intracellular pigment in 5% KOH, partial hyphae encrusted. Hypodermium well developed, yellowish to lightly brown intracellular pigmentation in 5% KOH. Clamp connections present.

Ecology and distribution: Gregarious in subtropical mixed forests. Known to inhabit Zhejiang Province, China. It may also distribute in Yunnan Province, China, and Japan, associated with *Monotropa hypopithys* and *Pseudotsuga japonica* according to the sequence information from GenBank.

Notes: *Thaxterogaster borealicremeolinus* is characterized by a yellowish-to-brownish, viscid pileus with a persistently wavy margin, whitish lamellae and stipe, large basidiospores, and a Northern Hemisphere distribution. Phylogenetically, *T. borealicremeolinus* is clustered with the species of sect. *Cremeolinae* and has formed a sister relationship with *T. dulciorum*, but the latter is only distributed in New Zealand, associated with *Nothofagus*, and the basidiospores are small, being <8 μm long [25]. Molecularly, the most closely related species are *T. cremeorufus* (94.16% similarity in ITS) and *T. nebulobrunneus* (94.15% similarity in ITS), in which *T. cremeorufus* is also distributed in the Southern Hemisphere, and associated with *Myrtaceae* trees and with smaller basidiospores < 10 μm long [26], while *T. nebulobrunneus* is a distinctly sequestrate species occurring in sub-alpine grassy woodland among *Eucalyptus* from Australia [27].

**Figure 3:**
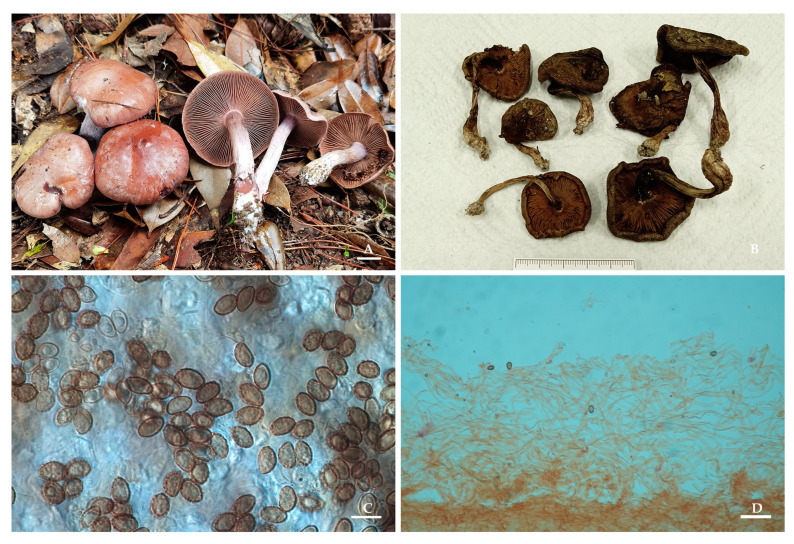
*Thaxterogaster rufopurpureus* (HMAS 287399). (**A**,**B**) Basidiomata; (**C**) Basidiospores; (**D**) Pileipellis in Congo Red reagent. Bars: (**A**) = 1 cm; (**C**) = 10 μm; (**D**) = 40 μm.

*Thaxterogaster rufopurpureus* M.L. Xie, Yi Li & W.F. Lin, *sp. nov.* Figure 3.

Fungal Names: FN 571636.

Etymology: The epithet ‘*rufopurpureus*’ refers to the color of the pileus.

Holotype: CHINA. Zhejiang Province, Longquan County, Pingnan Town, Dongshantou Village, in mixed forests, alt. 1180 m, 27°46′41″ N, 119°7′00″ E, 14 May 2023, *Wen-Fei Lin*, HMAS 287399.

Description. Pileus 2.5–4 cm in diam., hemispherical at first, later plano-convex, very finely innately fibrillose, viscid, greyish-purple to reddish-brown, turning deeper purple when bruised. Lamellae emarginated, moderately crowded, violet at first, greyish purple to brownish when mature, edges even, turning deeper purple when bruised. Stipe 2.5–6.5 cm long, 0.6–0.8 cm thick at the apex, clavate, violet-tinged at the apex, surface with fibrils, basal mycelium is white, turning deeper purple when bruised. Universal veil whitish. Context violet at the pileus and the upper part of the stipe, later becoming whitish with a violet tinge.

Basidiospores 9–10 × 5–6.5 μm, X_av._ = 9.3 × 6.1 μm, Q = 1.38–1.80, Q_av._ = 1.53, ellipsoid, moderately verrucose, indextrinoid. Basidia 29–46 × 8–14 μm, clavate, four-spored, hyaline or with yellowish intracellular pigment in 5% KOH. Lamellar edges fertile, with clavate sterile cells, 11–33 × 4–8.5 μm, thin-walled, hyaline in 5% KOH. Pileipellis duplex: epicutis of gelatinous, 100–230 µm thick, hyphae 2–7 μm wide, hyaline in 5% KOH, smooth. Hypodermium well developed, yellowish to lightly brown intracellular pigment in 5% KOH. Clamp connections present.

Ecology and distribution: Gregarious in subtropical mixed forests. Known to inhabit Zhejiang Province, China.

Notes: *Thaxterogaster rufopurpureus* is characterized by a greyish-purple to reddish-brown pileus of viscid, turning deeper purple when bruised at any part, moderately verrucose basidiospores, and a subtropical habitat. In phylogenetic terms, *T. rufopurpureus* formed a separate lineage in the sect. *Purpurascentes*, but its relationship with other species in this section is unclear. Molecularly, the most closely related species is *T. argyrionus* (Danks, T. Lebel & Vernes) Niskanen & Liimat. (95.27% similarity in ITS), a sequestrated species distributed in Australia.

**Figure 4:**
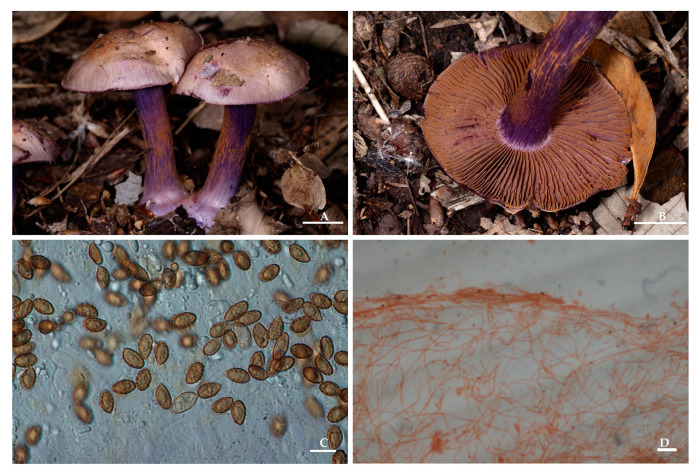
*Thaxterogaster sinopurpurascens* (HMAS 287400). (**A**,**B**) Basidiomata; (**C**) Basidiospores; (**D**) Pileipellis in Congo Red reagent. Bars: (**A**,**B**) = 1 cm; (**C**) = 10 μm; (**D**) = 50 μm.

*Thaxterogaster sinopurpurascens* M.L. Xie, Yi Li & JiaRui Guo, sp. nov. Figure 4.

Fungal Names: FN 571637.

Etymology: The epithet ‘*sinopurpurascens*’ refers to the affinity for *T. purpuracsens* and the type locality in China.

Holotype: CHINA. Jiangsu Province, Xuanwu District, Linggu Scenic Spot, in mixed forest, alt. 85 m, 32°3′27″ N, 118°51′49″ E, 28 June 2023, *Jia-Rui Guo*, HMAS 287400.

Description: Pileus 2–7 cm in diam., hemispherical at first, later plano-convex, innately fibrillose, glutinous, greyish-orange to brown, usually with a violet tinge, especially when young, with a paler margin, surface turning deeper purple when bruised. Lamellae emarginated, moderately crowded, violet at first, brownish-violet when mature, edges sometimes uneven, turning deeper purple when bruised. Stipe 2.2–6 cm long, 0.4–1 cm thick at the apex, marginate bulb at the base, ranging up to 2 cm, violet, surface with violet fibrils, turn deeper purple when bruised, basal mycelium purple tinge. Universal veil violet. Context violet at first, later whitish with a violet tinge.

Basidiospores 8–11(14) × 5–6(7) μm, X_av._ = 8.9–10.4 × 5.4–5.8 μm, Q = (1.38)1.45–1.91(2.80), Q_av._ = 1.63–1.81, ellipsoid, moderately verrucose, indextrinoid. Basidia 22.5–38 × 7–11 μm, subcylindrical to clavate, two- or four-spored, hyaline or with lightly yellowish intracellular pigment in 5% KOH. Lamellar edges fertile, with cylindrical to clavate sterile cells, 15–24 × 5–9 μm, thin-walled, hyaline in 5% KOH. Pileipellis duplex: epicutis of strongly gelatinous, 250–600 µm thick, hyphae 3–9 μm wide, the upper part hyaline to lightly violet in 5% KOH, the lower part yellowish-brown, smooth. Hypodermium well developed, yellowish to lightly brown intracellular pigment in 5% KOH. Clamp connections present.

Ecology and distribution. Gregarious in subtropical deciduous or mixed forests. Known to inhabit Chongqing City, Jiangsu Province, and Sichuan Province, China. It also inhabits Yunnan Province, China, according to the sequences.

Additional specimens examined. CHINA. Chongqing City, Bishan District, Jinjianshan Forest Park, in mixed forest, 13 June 2023, *Huan Wang*, HMAS 287401. Sichuan Province, Linshui County, Shiyong Town, Shihechang Village, in deciduous forest, alt. 420 m, 30°17′22″ N, 107°8′27″ E, 15 June 2023, *Yi-Ni Chen*, HMAS 287402, 21 June 2023, *Yi-Ni Chen*, HMAS 287403, Xuanhan County, 26 June 2023, *Xian-Mei Yang*, HMAS 287404.

Notes: *Thaxterogaster sinopurpurascens* is a kind of wild edible fungus characterized by a greyish-orange to brown pileus, violet lamellae, stipe, context, and universal veil, turning deeper purple when bruised at any part, a distinctly marginate bulb at the base of the stipe, and ellipsoid basidiospores. *Thaxterogaster sinopurpurascens* is easily confused with *T. indopurpurascens* and *T. purpurascens* morphologically. In phylogenetics, the new species also clustered with *T. indopurpurascens*, *T. purpurascens*, and *T. shoreae*. In addition, molecularly, the most closely related species are *T. indopurpurascens* (98.51% similarity in ITS) and *T. purpurascens* (98.18% similarity in ITS). However, *T. indopurpurascens* is a pale bluish-grey when young and has a roundish marginated bulb at the base of the stipe [28], *T. purpurascens* usually has smaller basidiopores, amounting to 7.2–8.8 × 4.5–5.3 µm [29], and *T. shoreae* usually has a roundish marginated bulb at the base of the stipe and is associated with *Shorea robusta* in India [30].

## 4. Discussion

In sect. *Cremeolinae*, only five known species were reported across the globe before this study, and all the species are distributed in the Southern Hemisphere [31]. So, *T. borealicremeolinus* is the first Northern-Hemisphere-distributed species in this section. Two mycorrhizal sequences, in which JQ396472 is from *Monotropa hypopithys* in Yunnan, China, and AB807927 is from *Pseudotsuga japonica* in Japan, which were clustered together with our specimen, were treated as new species. *Thaxterogaster* is usually associated with species of *Fagaceae*, *Betulaceae*, *Tilia*, and *Pinaceae* in the Northern Hemisphere and associated with *Nothofagaceae* in the Southern Hemisphere [4]. The sequence JQ396472 shows that some species of *Monotropoideae*, a group of non-autotrophic plants, may also be a host plant of *Thaxterogaster* species [32].

In sect. *Purpurascentes*, two new species formed separate clades in the phylogenetic trees, and their morphological and ecological characteristics also approved their novelty. One specimen from Yunnan province, China (i.e., HKAS122529), was identified as *T. purpurascens*; here, we treated it as misidentified due to the ITS sequence clustered with *T. sinopurpurascens* and as distinct from the European *T. purpurascens*. The latter has been widely reported in China in the past, but so far, we have not found any exact specimens to confirm its Chinese distribution.

Based on our work in recent years, we have confirmed twelve known *Thaxterogaster* species distributed in China [10,11]. But there are still ten *Thaxterogaster* species reported to inhabit China in previous studies that lacked molecular data, and some of them also did not provide morphological features. The lack of intensive research on *Thaxterogaster* has led to a deficit of knowledge of the true species diversity of this genus in China. Here, we present the first research article focusing on the genus *Thaxterogaster* from China. Moreover, the basal lineage of *Cortinariaceae* [4,31] also shows the evolutionary importance of *Thasterogaster* in *Cortinariaceae*. So, this genus in China needs further study in the future.

## Figures and Tables

**Table 1:** Voucher information and their GenBank accession numbers for ITS sequences.

Species	Voucher	Specimen Status	GenBank Accession no.	Locality
*Thaxterogaster anomalo-ochrascens*	PC: RH2805	Holotype	MH846269	France
*T. argenteolilacinus*	M: MM48/752	Holotype	MH846277	Austria
*T. argyrionus*	MD162		GQ890313	Australia
*T. argyrionus*	MEL: 2331642	Holotype	NR152999	Australia
*T. armenicorius*	KS-CO1865	Holotype	KM504515	France
***T. borealicremeolinus***	**HMAS 287398**	**Holotype**	**OR395228**	**China**
***T. borealicremeolinus***	**LY418**		**OR395363**	**China**
*T. borealicremeolinus*	38-1A		JQ396472	China
*T. borealicremeolinus*	Sa17-501		AB807927	Japan
*T. burlinghamiae*	DBB37303	Holotype	KX768115	USA
*T. caesiolamellatus*	PC: PML4905	Holotype	NR157955	France
*T. caesiophylloides*	H: 6029792	Holotype	NR130303	Finland
*T. chalybeus*	PDD: 73146	Holotype	NR157871	New Zealand
*T. chalybeus*	CO1342		AY669613	Germany
***T. rufopurpureus***	**HMAS 287399**	**Holotype**	**OR395229**	**China**
*T. cinereoroseolus*	MEL: 2331646	Holotype	NR153000	Australia
*T. collocandoides*	CR502		MK472686	Luxembourg
*T. collocandoides*	PC: PML5087	Holotype	NR130210	France
*T. cremeolina*	PDD: 70506	Holotype	NR157889	New Zealand
*T. cremeorufus*	PDD: 94056	Holotype	NR153064	New Zealand
*T. cremeorufus*	PDD: 72649		KT833622	New Zealand
*T. dovrensis*	O: TEB112-80	Holotype	NR160640	Norway
*T. dulciorum*	PDD: 78797	Holotype	NR157898	New Zealand
*T. dulciorum*	PDD: 107708		KT875195	New Zealand
*T. frondosomultiformis*	TG2000-218	Holotype	KM504516	Italy
*T. fulvo-ochrascens*	PC: RH314	Holotype	MH846266	France
*T. glaucocyanopus*	G: 5034	Holotype	MH846274	France
*T. indopurpurascens*	KCS: 2442	Holotype	NR173956	India
*T. indopurpurascens*	KCS: 2529		MW135430	India
*T. iringa*	PDD: 73135	Holotype	NR120131	New Zealand
*T. kaimanawa*	PDD: 101872		KJ635219	New Zealand
*T. kaimanawa*	PDD: 73133	Holotype	NR157891	New Zealand
*T. malachioides*	TEB340-16		MH923033	Sweden
*T. melleicarneus*	H: IK01-053	Holotype	KF732577	Estonia
*T. mendax*	PC: AB07-10-162	Holotype	NR153019	France
*T. multiformis*	S: F44806	Neotype	KF732350	Sweden
*T. nebulobrunneus*	MEL: 2331648	Holotype	NR152995	Australia
*T. occidentalis*	MICH: 10382	Holotype	NR130234	USA
*T. pallidirimosus*	H: 6035694	Holotype	KF732578	Finland
*T. pallidoriederi*	BOZ: Bellu 30-09-2011	Holotype	NR160639	Italy
*T. porphyropus*	S: F47381	Neotype	NR130246	Sweden
*T. porphyropus*	SAT-16-237-03		MW597131	USA
*T. purpurascens*	H: IK98-2121	Neotype	NR130252	Sweden
*T. purpurascens*	M: 0275842		KT159212	Portugal
*T. purpurascens*	TUB 019710		KJ421138	Austria
*T. rhipiduranus*	PDD: 72617		MH101624	New Zealand
*T. rhipiduranus*	PDD: 88269	Holotype	NR157902	New Zealand
*T. riederi*	BOZ: Bellu 12-08-2012		MH923057	Italy
*T. riederi*	TEB141-10		MH923056	Sweden
*T. rufoallutus*	PC: PML635	Holotype	KF732413	France
*T. shoreae*	AGJH-017		OP473978	India
*T. shoreae*	AGDC_21-04	Holotype	OP473976	India
***T. sinopurpurascens***	**HMAS 287400**	**Holotype**	**OR395230**	**China**
***T. sinopurpurascens***	**HMAS 287401**		**OR395231**	**China**
***T. sinopurpurascens***	**HMAS 287402**		**OR395232**	**China**
***T. sinopurpurascens***	**HMAS 287403**		**OR395233**	**China**
***T. sinopurpurascens***	**HMAS 287404**		**OR395234**	**China**
*T. sinopurpurascens*	HKAS 122529		ON794354	China
*T.* sp.	H0920		DQ328090	Australia
*T. subinops*	PC: PML5119	Holotype	NR157964	France
*T. submagellanicus*	MEL: 2305432	Isoparatype	NR173466	Australia
*T. talimultiformis*	UPS: AT2004096	Holotype	NR130306	Sweden
*T. talus*	S: F44793	Neotype	KF732457	Sweden
*Phlegmacium boreicyanites*	S: CFP931	Holotype	KF732296	Sweden
*P. cyanites*	UPS: AT2005069	Neotype	KF732355	Sweden

Newly generated sequences were marked in bold.

## Data Availability

All newly generated sequences were deposited in GenBank (https://www.ncbi.nlm.nih.gov/genbank/; accessed on 4 August 2023). All new taxa were linked with Fungal Name (https://nmdc.cn/fungalnames/; accessed on 5 August 2023).
